# Targeted Modulation of Zn Deposition Behavior toward High-Performance Stable Zn Anodes

**DOI:** 10.1007/s40820-026-02350-1

**Published:** 2026-08-25

**Authors:** Peng Zhang, Min Wang, Yang Yang, Juan Tian, Man Huang, Lili Chen, Jingang Wang, Caixia Xu, Dedong Jia, Hua Tan, Hong Liu, Dongliang Chao, Weijia Zhou

**Affiliations:** 1 Institute for Advanced Interdisciplinary Research (iAIR), School of Chemistry and Chemical Engineering, University of Jinan, Jinan, 250022 People’s Republic of China; 2 Laboratory of Advanced Materials, Shanghai Key Laboratory of Molecular Catalysis and Innovative Materials, College of Smart Materials and Future Energy, Fudan University, Shanghai, 200433 People’s Republic of China; 3 State Key Laboratory of Crystal Materials, Shandong University, Jinan, 250100 People’s Republic of China; 4 School of Chemistry and Chemical Engineering, Anhui University of Technology, Maanshan, 243002 People’s Republic of China

**Keywords:** Aqueous zinc-ion batteries, Zn anode design, Zincophilicity, Lattice matching, 3D structural design

## Abstract

Dendrite growth and parasitic side reactions at the Zn metal anode remain critical obstacles to the commercialization of aqueous zinc-ion batteries (AZIBs). Zn anode design regulates Zn deposition behavior from its thermodynamic and kinetic roots and is therefore recognized as a pivotal strategy to address the above bottlenecks. However, Zn deposition evolves through a complex multistage nucleation-growth process, and the structure–property relationship between the anode designs and practical Zn deposition behavior remains to be elucidated. This review focuses on Zn deposition substrate and functional interphase engineering, systematically elucidates the microscopic mechanisms and key regulatory targets of Zn deposition at each stage, and establishes a multidimensional regulation framework covering atomic tuning, interfacial modification, and spatial confinement. Based on this framework, we summarize three core design strategies, namely zincophilic regulation, lattice-matched effects, and three-dimensional structural engineering, and provide in-depth elucidation of the working mechanism and structure-property relationship of each strategy. By correlating these fundamental principles with the latest research advances in the field, we highlight the paradigm shift from empirical trial and error to rational material design. Finally, we outline the prevailing challenges and future research directions for Zn anode design, aiming to provide actionable insights for the development of advanced dendrite-free Zn anodes toward high-performance AZIBs.

## Highlights

- Zn anodes modification strategies are systematically summarized, with emphasis on the full-sequence nucleation and growth of Zn electrodeposition.
- A full-chain multi-scale regulatory framework is established, analyzing the working mechanisms, design principles, and state-of-the-art advances of three core complementary strategies for Zn anode design.
- Critical challenges in practical anode engineering are outlined, the paradigm shift from empirical trial-and-error to rational material design is highlighted, and forward-looking research directions for commercial aqueous zinc-ion batteries are proposed.

## Graphical abstract

**Figure:**
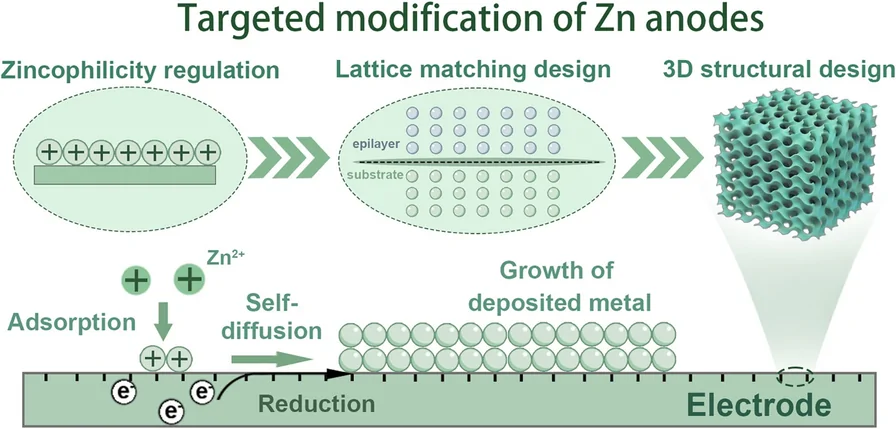

## Introduction

**Fig. 1:**
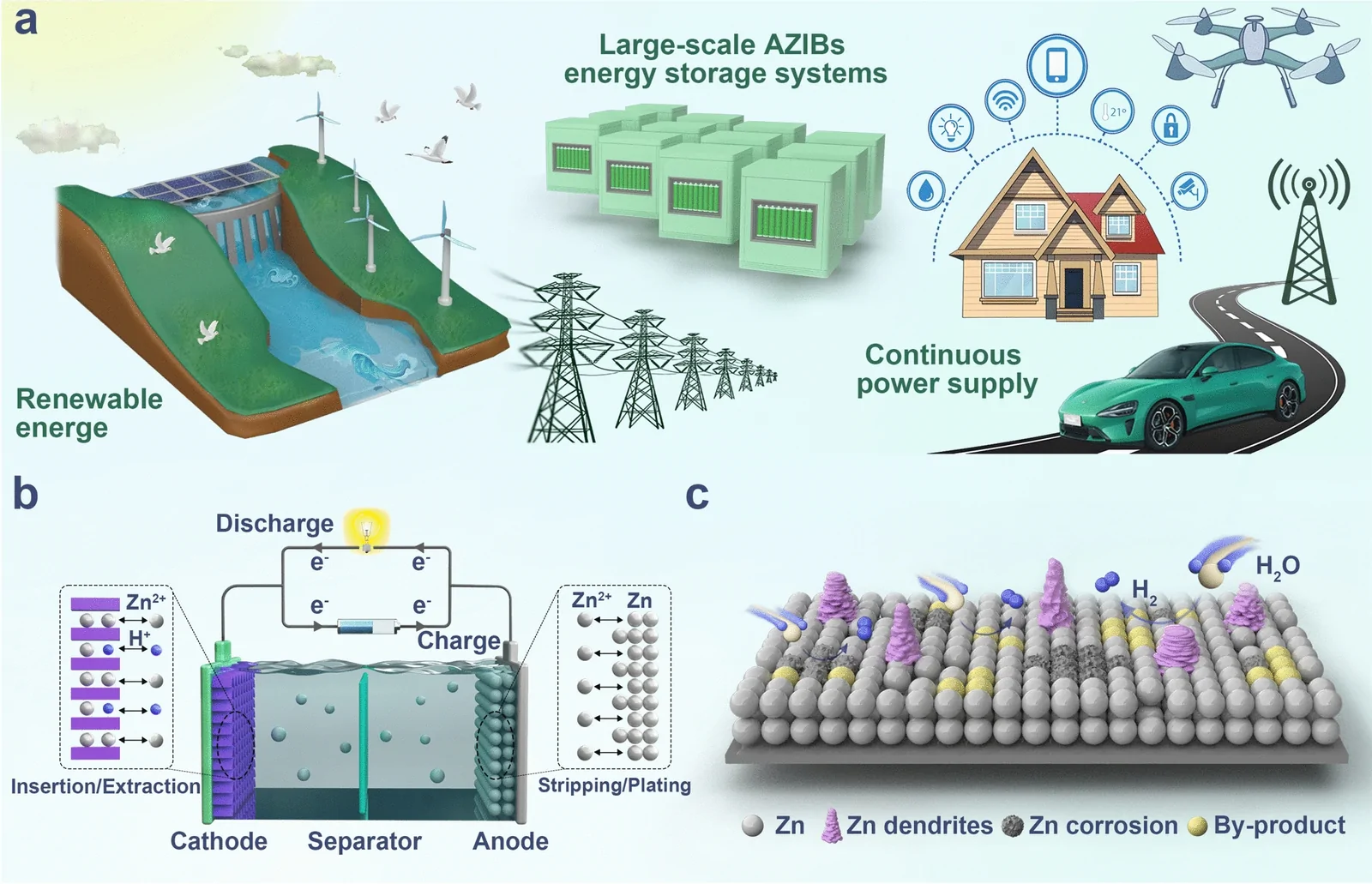
Challenges and structural characteristics of Zn anodes in AZIBs. **a** Schematic illustration of AZIBs implementation in grid-scale renewable energy storage systems. **b** Working mechanism of a typical AZIB. **c** Key challenges affecting Zn anode performance, including dendritic growth, hydrogen evolution, and surface passivation

The worldwide shift toward renewable energy has accelerated the deployment of solar, wind, and hydropower, yet their intermittency creates a critical need for reliable, grid-scale energy storage systems (Fig. 1a, left panel) [1–4]. However, the fluctuating nature of these renewables poses substantial obstacles to grid stability [5–7]. Within this context, aqueous zinc-ion batteries (AZIBs) are regarded as a promising replacement for lithium-ion systems, owing to their intrinsic safety provided by nonflammable aqueous electrolytes, low cost, and environmental compatibility arising from the abundance and nontoxicity of Zn resources [8–13]. Furthermore, Zn metal offers a high theoretical capacity (820 mAh g^–1^ or 5855 mAh cm^–3^) and a suitable redox potential (–0.76 V vs. standard hydrogen electrode) [14–18], providing crucial advantages for constructing high-energy-density storage systems (Fig. 1). These attributes collectively endow AZIBs with broad application potential across diverse fields such as smart grids, autonomous vehicles, and aerospace (Fig. 1a, right panel). Nevertheless, the practical performance and commercialization of AZIBs remain limited by the intrinsic defects of Zn anodes during cycling, which directly compromise long-term electrochemical stability [19–21].

AZIBs operate on a “rocking-chair” mechanism, wherein energy storage and release are realized through the reversible insertion/extraction of Zn^2+^ ions within the cathode, coupled with the reversible dissolution/deposition of Zn at the anode (Fig. 1b) [22–26]. Consequently, the reversible Zn plating and stripping behavior on the anode dictates the long-term cycling life of AZIBs [27–32]. Ideally, Zn deposition would proceed via a layer-by-layer growth mode that follows its native crystal lattice, yielding a densely packed, uniform morphology [33]. In practical battery operation, however, Zn anodes inevitably suffer from three closely interrelated failure modes (Fig. 1c). Primarily, inhomogeneous electric-field distribution and insufficient nucleation sites lead to uneven Zn deposition and dendrite formation. The dendritic growth perforates the separator, inducing internal short-circuiting of the cell [34–38]. Secondarily, the parasitic hydrogen evolution reaction (HER) irreversibly depletes protons and lowers Coulombic efficiency, while also inducing local pH fluctuations that accelerate Zn corrosion [39–42]. The buildup of gaseous hydrogen further elevates internal pressure, risking electrolyte leakage or catastrophic cell failure [43]. Thirdly, electrically insulating passivation byproducts (e.g., Zn_4_SO_4_(OH)_6_·*x*H_2_O) generated by complex interfacial reactions passivate the anode surface and severely degrade cycling stability [44–46]. Uniform and compact Zn deposition suppresses dendrite-induced short circuits and dead Zn formation, minimizes parasitic reactions associated with enlarged active surface area, and consequently enables AZIBs with lower polarization, higher Coulombic efficiency, and prolonged cycle life.

Zn anode design represents a highly promising approach to address the aforementioned challenges. It targets the root causes of anode failure, which are the inherent inhomogeneities in nucleation site distribution, interfacial reaction kinetics, and mass transport during Zn plating/stripping. Specifically, it directly modulates the physicochemical properties of the electrode itself by tailoring surface/interface characteristics, crystallographic texture and three-dimensional (3D) topological architecture. This approach endows the anode with intrinsic capabilities to inhibit dendrite formation and side reactions, thus offering a more sustainable pathway for improving the performance of AZIBs [47–50]. Recently, substantial progress has been made in Zn anode engineering toward these objectives. However, the field still lacks a systematic mechanistic understanding of the complex, multistage, and multiscale evolutionary behaviors of the Zn deposition. Most reported Zn anode engineering methods center on targeted regulation of individual steps of Zn deposition, such as diffusion, adsorption, nucleation, and growth, thereby failing to achieve synergistic regulation across the full nucleation-growth process of Zn deposition. Therefore, proceeding from the fundamental nature of the Zn plating process, this review focuses on the Zn deposition substrate and functional interface engineering to optimize Zn deposition behavior, thereby laying the groundwork for constructing highly stable Zn metal anodes.

Herein, we systematically review the design principles and optimization strategies for Zn anodes, focusing on the regulation of the Zn deposition process to address the critical knowledge gap between the complex multistage nucleation-growth process and deposition behavior. By integrating atomic-level site engineering, interfacial lattice matching, and microscopic spatial confinement, we first deconstruct the microscopic mechanisms and core regulatory targets of each stage during Zn deposition. Then systematically discuss three core design strategies (zincophilic regulation, lattice‑matched effects, and 3D structural engineering) under this framework, with comprehensive analysis of their working mechanisms and structure-property relationships. We further highlight the paradigm shift from empirical trial and error to rational material design in this field and finally outline the prevailing challenges and forward-looking research directions for Zn anode modification. Overall, this review aims to establish fundamental structure-property relationships and design criteria for suppressing dendrite growth and parasitic reactions, thereby providing theoretical guidance and scientific foundations for advancing high-stability Zn metal anodes toward practical applications in AZIBs.

## Fundamental Mechanisms of Zn Deposition and Targeted Regulation of Zn Anodes

The electrochemical deposition of Zn metal directly determines the cycling reversibility, Coulombic efficiency, and structural integrity of AZIBs. Consequently, establishing an in‑depth comprehension of the fundamental reduction mechanisms and developing targeted modulation strategies have emerged as the core priority of ongoing research. Rational structural design is of decisive importance for facilitating homogeneous Zn plating and mitigating dendrite formation. The fundamental rationale behind this strategy lies in the deep insight and precise regulation of each sequential stage of Zn plating. This chapter systematically deconstructs the Zn deposition process, clarifies the origin of deposition inhomogeneity and failure, and builds a full-chain multiscale regulatory framework that runs through the entire review.

**Fig. 2:**
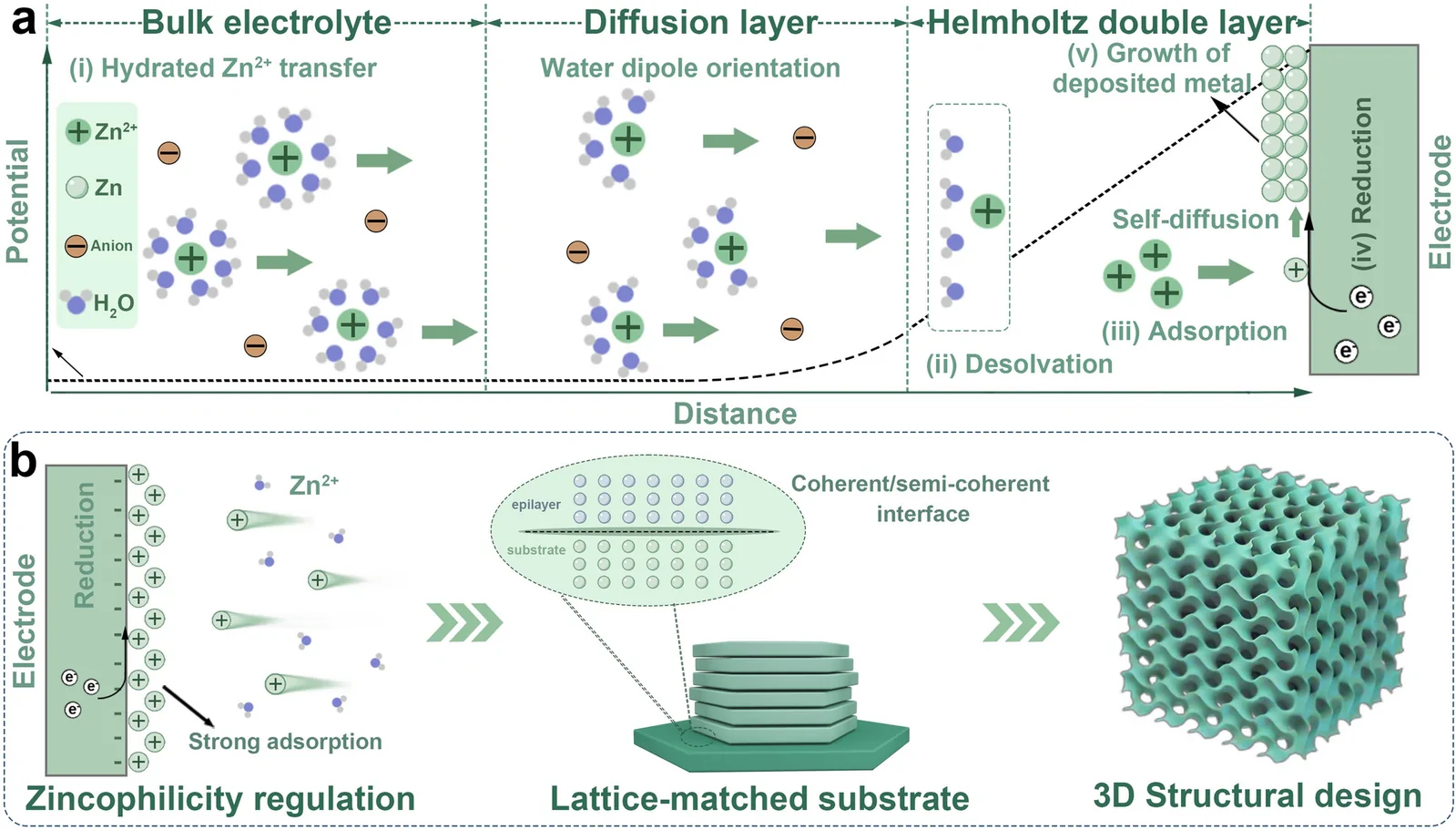
Electrodeposition behavior of Zn in aqueous electrolytes. **a** Schematic illustration of the multistage Zn electrodeposition process in mildly acidic/neutral electrolytes, depicting the sequential steps from bulk transport to interfacial growth. Reproduced with permission [51]. Copyright 2022, Royal Society of Chemistry. **b** Schematic of Zn anode design encompassing three core regulation strategies: zincophilicity modulation, lattice matching engineering, and 3D structural regulation

In the mildly acidic and/or neutral electrolytes that dominate the current research of AZIBs, the Zn deposition process undergoes five sequential and interconnected stages, each directly affecting the final deposition morphology and interfacial stability (Fig. 2a). These stages include: (i) migration of hydrated Zn^2+^ ions from the bulk electrolyte to the Helmholtz double layer; (ii) desolvation of hydrated Zn^2+^ ions at the Helmholtz double layer, the key kinetic step for charge transfer; (iii) adsorption of desolvated Zn^2+^ ions onto the anode surface; (iv) electroreduction of adsorbed Zn^2+^ ions to Zn nuclei; and (v) growth of Zn nuclei into bulk metallic deposits [51–55].

The final morphological evolution and crystallographic habit of Zn electrodeposits are predominantly governed by the nucleation (stages iii and iv) and subsequent growth (stage v) processes, which represent the core regulatory targets of anode design (Fig. 2b). Thermodynamically, Zn nucleation follows classical heterogeneous nucleation theory, where a low nucleation energy barrier favors uniform instantaneous nucleation, the prerequisite for dendrite-free deposition. Kinetically, Zn growth behavior is determined by the substrate crystallographic structure, interfacial Zn^2+^ flux, and local electric field distribution, which collectively dictate the formation of compact deposits or disordered dendrites. Based on the full-sequence deposition mechanism and core regulatory targets clarified above, this review establishes a multiscale regulatory framework for Zn anode design with three core strategies. These approaches can be grouped into three complementary categories based on their primary mechanisms and the scales at which they operate. The first strategy is zincophilicity modulation, which optimizes the physicochemical properties of the Zn substrate to enhance the binding affinity with Zn atoms, thus reducing the adsorption energy and nucleation barrier of Zn^2+^ ions and facilitating uniform nucleation. The second strategy is lattice-matching engineering, which leverages crystallographic orientation to guide the preferential orientation of growing grains and mitigates interfacial stress during growth, facilitating the formation of smooth and compact deposition layers. The third strategy is structural regulation, which homogenizes the local electric field and Zn^2+^ flux, alleviates concentration polarization, and accommodates volumetric expansion during repeated plating/stripping cycles. These three strategies synergistically regulate the Zn deposition behavior from the atomic, interfacial to microscale, and the subsequent chapters systematically discuss their working mechanisms, structure-property relationships, and state-of-the-art research advances respectively.

## Zincophilicity Regulation for Uniform Zn Nucleation

The nucleation process of Zn^2+^ directly determines the initial distribution of Zn deposits and is the core regulatory target to suppress dendrite initiation from the source. Zincophilicity modulation, as the first core strategy of the multiscale regulation framework, achieves targeted optimization of the Zn^2+^ adsorption and nucleation process by tuning the inherent physicochemical properties of the Zn matrix. This chapter systematically elucidates the fundamental mechanisms of zincophilicity in regulating Zn electrochemical nucleation and summarizes the latest modification strategies for optimizing zincophilicity, with in-depth analysis of their structure-property relationships in stabilizing Zn anodes.

### Fundamentals of Zincophilicity

**Fig. 3:**
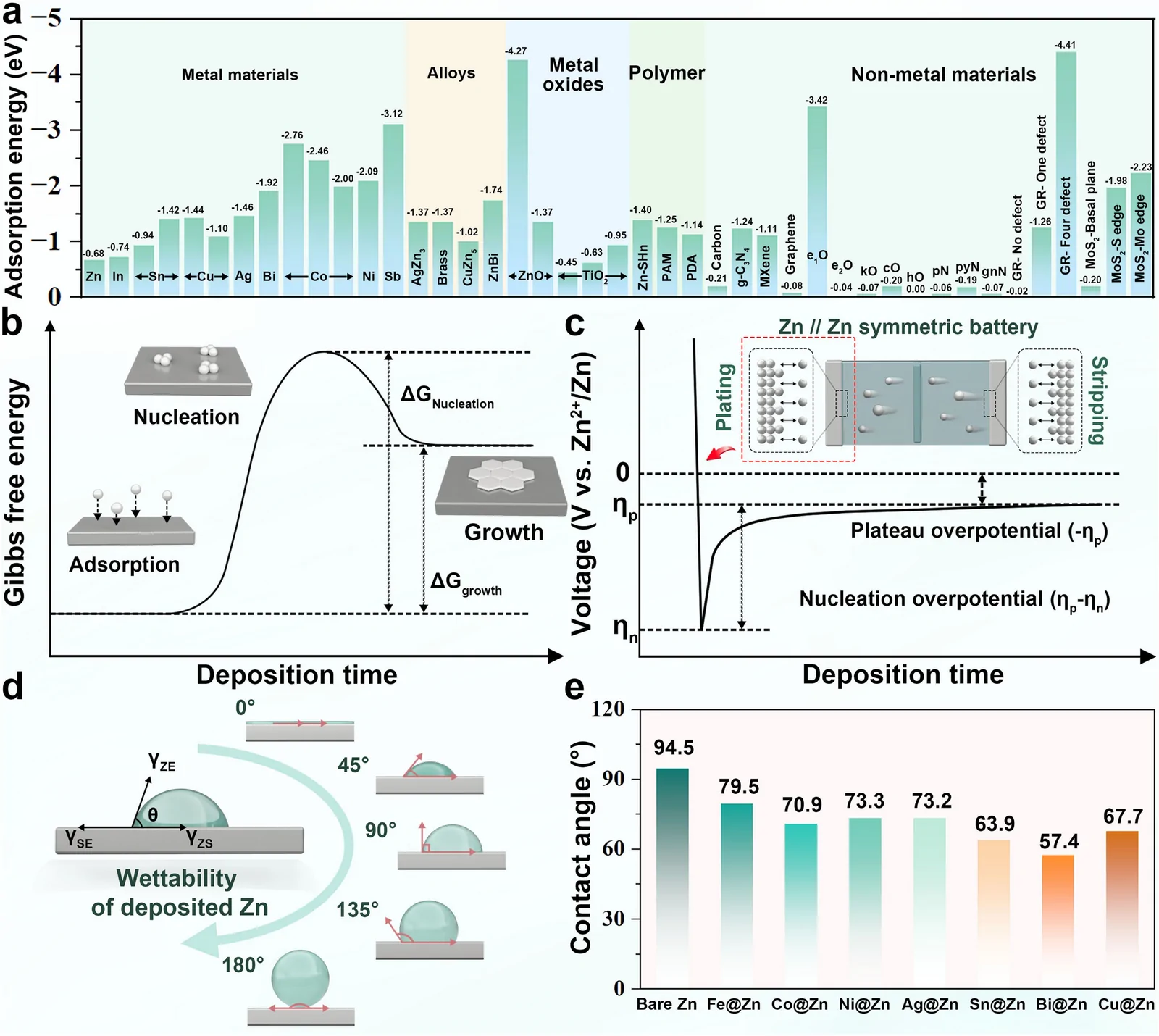
The role of zincophilicity in modulating Zn nucleation behavior. **a** The binding energy between Zn and common substrate materials. Reproduced with permission [61]. Copyright 2023, American Chemical Society. **b** The Gibbs free energy diagram and **c** voltage profile for initial Zn nucleation processes. Reproduced with permission [52]. Copyright 2024, Royal Society of Chemistry. **d** The wettability of electrolyte on substrate. Reproduced with permission [71]. Copyright 2024, Wiley–VCH. **e** The contact angles between Zn and other metals. Reproduced with permission [58]. Copyright 2023, Wiley–VCH

Zincophilicity describes the intrinsic binding affinity of a substrate toward Zn atoms and is crucial for regulating initial nucleation behavior and interfacial adhesion during Zn electrodeposition [49, 56, 57]. Appropriate zincophilicity enhancement reinforces the interfacial adhesion between deposited Zn atoms and the substrate, promotes uniform nucleation, improves interfacial stability, and suppresses dendrite formation [58–60]. The binding energy (${E}_{bind}$) between Zn atoms and the substrate is the core quantitative parameter to evaluate zincophilicity and can be calculated as Eq. (1) [61]:

1 $$\begin{array}{c}{E}_{bind} = {E}_{total} - {E}_{Zn} - {E}_{substrate}\end{array}$$

where ${E}_{total}$, ${E}_{Zn}$, and ${E}_{substrate}$ are the total energy of the Zn-adsorbed substrate system, the energy of an isolated Zn atom, and the energy of the substrate, respectively [52, 62]. A relatively positive *E*_bind_ indicates zincophobic behavior, whereas values substantially more negative than the Zn-Zn cohesive energy benchmark (−0.68 eV) confirm zincophilic character [63, 64]. Generally, more negative binding energies correlate with enhanced zincophilicity. Systematic theoretical calculations and experimental validations have established a clear zincophilicity database for common electrode materials, as summarized in Fig. 3a. These zincophilic materials mainly include metallic elements (e.g., In, Sn, Cu, Ag, Bi, Co, Ni, and Sb) [6, 41, 58, 65, 66], alloy phases (e.g., AgZn_3_, brass, and CuZn_5_) [41, 65, 66], metal oxides (e.g., ZnO and TiO_2_) [65, 67], functional polymers (e.g., Zn-SHn, polyacrylamide (PAM), and polydopamine (PDA)) [66], as well as nonmetallic materials (e.g., g-C_3_N_4_ and graphene) [61, 68, 69]. These comprehensive investigations establish a fundamental materials database that enables the rational design of stabilized Zn anode substrates through adsorption energy optimization.

The initial stage of Zn electrodeposition follows classical heterogeneous nucleation theory, which requires overcoming a nucleation energy barrier (${\Delta G}_{nucleation}$) associated with solid-phase formation (Fig. 3b) [66, 70]. This barrier manifests experimentally as a nucleation overpotential in the deposition voltage profile. Typically, a characteristic signature is an initial rapid voltage drop to the nucleation potential (*η*_n_), followed by a recovery to a stable plating plateau (*η*_p_). The potential difference (*η*_p_−*η*_n_) quantifies the nucleation overpotential (Fig. 3c) [34]. Serving as a heterogeneous nucleation platform during Zn electrodeposition, the substrate with high zincophilicity can reduce ${\Delta G}_{nucleation}$ and the overpotential, which facilitates uniform Zn nucleation and accelerates Zn deposition kinetics [52, 72]. Furthermore, according to classical heterogeneous nucleation theory, the nucleation barrier $\Delta {G}_{het}^{*}$ is determined by [70, 71, 73, 74]:

2 $$\begin{array}{c}\Delta {G}_{het}^{*} = S\left(\theta \right)\Delta {G}_{hom}^{*}\end{array}$$

3 $$\begin{array}{c}S\left(\theta \right)=\frac{\left(2 + \mathit{cos}\theta \right){\left(1 - \mathit{cos}\theta \right)}^{2}}{4}\end{array}$$

4 $$\begin{array}{c}\mathit{cos}\theta =\frac{{\gamma}_{SE} - {\gamma}_{ZS}}{{\gamma}_{ZE}}\end{array}$$

where $\Delta {G}_{het}^{*}$ and $\Delta {G}_{hom}^{*}$ correspond to the heterogeneous and homogeneous nucleation barrier, respectively. ${\gamma}_{SE}$, ${\gamma}_{ZS}$ , and ${\gamma}_{ZE}$ represent the interfacial energies between substrate–electrolyte, Zn nucleus–substrate, and Zn nucleus–electrolyte, respectively; and *θ* is the contact angle reflecting the wettability of deposited Zn nuclei on the substrate (Fig. 3d). The contact angle (*θ*) critically determines the nucleation mode [75]. As summarized in Fig. 3e, different Zn-based electrodes exhibit distinct contact angles in ZnSO_4_ electrolyte [58, 71]. When *θ* approaches zero, the $\Delta {G}_{het}^{*}$ diminishes nearly to zero, facilitating spontaneous Zn nucleation through minimizing the interface formation energy [58]. Generally, higher zincophilicity lowers the ${\Delta G}_{nucleation}$ and the overpotential, thereby facilitating uniform instantaneous nucleation across the whole Zn anode. After uniform crystal nuclei are formed on the electrode surface, subsequent preferential crystal growth proceeds on these pre-formed nuclei rather than at random locations, which in turn enables uniform Zn plating.

Based on the fundamental mechanisms clarified above, various modification approaches have been developed to enhance the surface zincophilicity of Zn anodes, such as the incorporation of heterometallic sites, grafting of polar functional groups, and defect-engineered materials. These strategies achieve targeted regulation of Zn nucleation behavior via tuning the inherent physicochemical properties of the Zn matrix itself, without introducing additional inactive exogenous components that sacrifice full-cell energy density.

### Strategies for Surface Zincophilicity Optimization

**Fig. 4:**
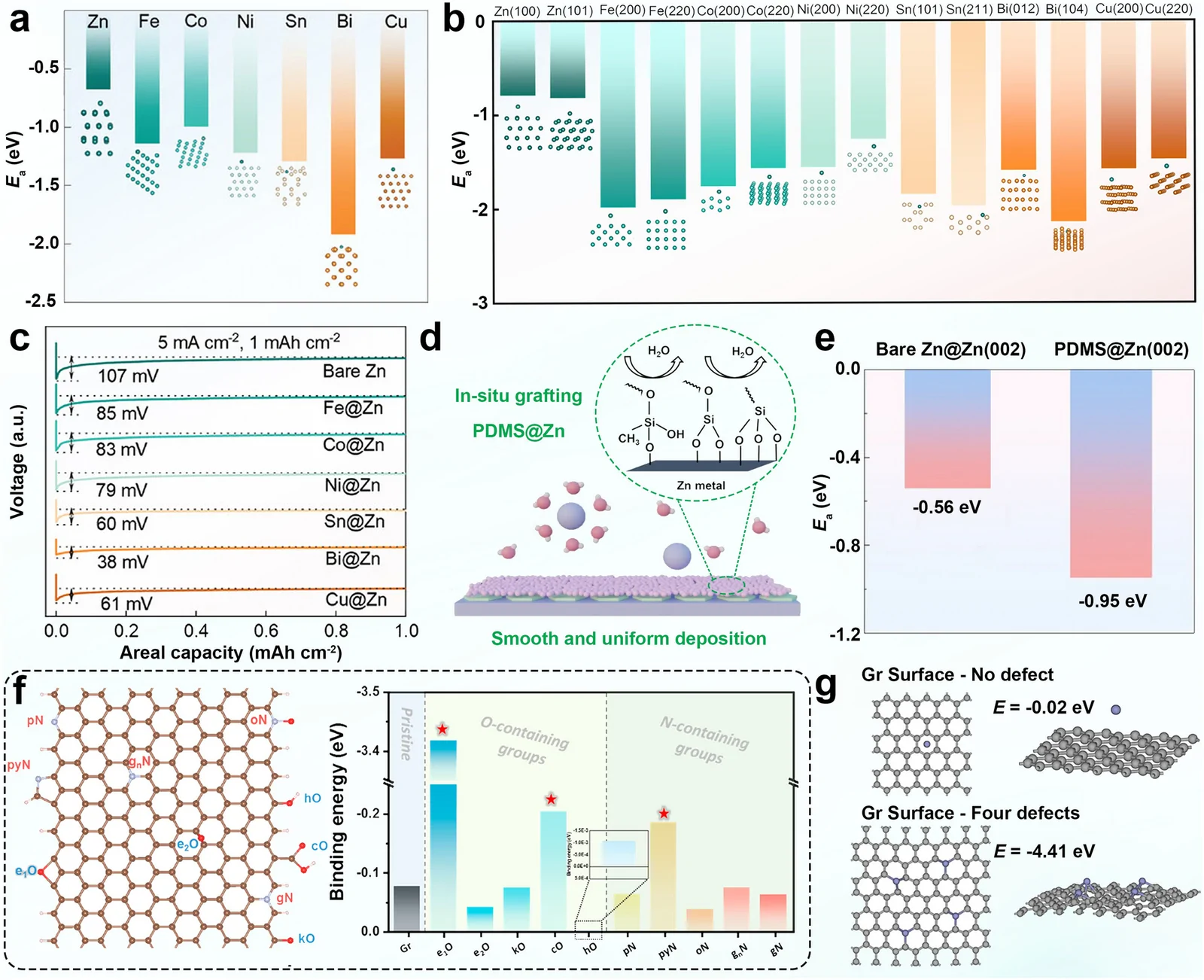
Modulation of zincophilicity to facilitate uniform Zn nucleation. **a** Calculated binding energies between Zn and representative substrate materials. **b** Adsorption energy (*E*_*a*_) and corresponding adsorption configurations of a Zn atom onto different crystal facets. **c** Polarization curves of Zn deposition at 5 mA cm^−2^ with an areal capacity of 1 mAh cm^−2^. Reproduced with permission [58]. Copyright 2023, Wiley–VCH. **d** Schematic illustration of the working mechanism of the PDMS@Zn anodes. **e** Calculated adsorption energy of Zn^2+^ on bare Zn and PDMS@Zn surfaces. Reproduced with permission [83]. Copyright 2025, Wiley–VCH. **f** Computational models of O-doped and N-doped graphene, along with a summary of calculated binding energies between Zn atoms and all possible configurations of pristine, O-doped, and N-doped graphene. Reproduced with permission [68]. Copyright 2022, American Chemical Society. **g** Binding energy comparison of pristine and defect-modified graphene (Gr) configurations. Reproduced with permission [84]. Copyright 2019, American Chemical Society

Recent advances have demonstrated that introducing heterometallic sites can effectively boost the zincophilicity of Zn anodes, which optimizes the interfacial binding energy toward Zn atoms, reduces the nucleation energy barrier, and promotes uniform Zn nucleation [76, 77]. Various zincophilic heterometallic elements have been explored for this purpose, including Au, Ag, In, Cu, Sn, and Ni [78, 79]. However, the zincophilicity and electrochemical performance of these heterometallic sites vary significantly, posing distinct challenges for their practical application in stable Zn anodes. Among the various heterometallic candidates, bismuth (Bi) stands out due to its superior zincophilicity. The robust interfacial bonding between the Bi substrate and Zn atoms is observed in Fig. 4a, b, with Bi exhibiting the lowest Zn binding energy compared to other metal candidates (i.e., Sn, Cu, Ni, Fe, Co, and Zn) [58]. Bi@Zn interfaces exhibit the smallest nucleation overpotential (38 mV), as shown in the polarization curves (Fig. 4c). This lower nucleation overpotential stems from the thermodynamically favorable Bi-Zn interaction, which boosts electrolyte wettability and suppresses HER. Consequently, the Bi@Zn heterometallic interface achieves over 4700 cycles at 10 mA cm^−2^, alongside a small and stable overpotential of only ≈55 mV. Beyond the zincophilicity of the selected heterometallic element, the crystal orientation of the heterometallic sites serves as another critical and tunable handle to further optimize Zn binding affinity and nucleation behavior. Given the facet-dependent Zn binding energies, deliberately exposing crystal facets with higher zincophilicity provides a direct route to regulate the Zn deposition mode. Zhang et al. demonstrated that selective exposure of the TiO_2_(001) crystal facet can effectively tune the zincophilicity, thereby enabling the design of high-performance Zn anodes with suppressed dendrite growth [80].

Covalent grafting of polar functional groups on the Zn matrix surface offers an additional feasible route to enhance zincophilicity. Recently, studies have demonstrated that organic polymers bearing abundant polar groups (e.g., −C−O −C, −C=O, −COO− , −COOH, −OH, −NH_2_, and −OCH_3_) exhibit excellent zincophilicity [57, 81, 82]. These polar groups can coordinate with Zn^2+^ to regulate its nucleation behavior, thereby facilitating homogeneous Zn deposition. In-situ grafting of hydroxyl-containing poly(dimethylsiloxane) (PDMS) onto the Zn anode surface is achieved through Si–O–Zn bonds (Fig. 4d). Density functional theory (DFT) calculations show that the PDMS@Zn surface delivers a Zn^2+^ adsorption energy of −0.95 eV, considerably lower than the −0.56 eV on bare Zn, which originates from the strong binding energy between the PDMS hydroxyl groups and Zn^2+^ on the (002) crystal plane (Fig. 4e). This strong Zn^2+^−PDMS interaction imparts superior zincophilicity and consequently promotes uniform Zn deposition [83].

Zincophilicity is also inherently governed by the surface chemical state and defect structure of the substrate. Defect engineering and heteroatom doping of the substrate can effectively create abundant zincophilic active sites, optimizing the Zn adsorption behavior and nucleation mode [85, 86]. For example, pristine graphene composed of sp^2^-hybridized carbon exhibits zincophobicity, arising from its weak binding interaction with Zn atoms. Conversely, the deliberate introduction of heteroatom dopants and/or the generation of point/edge defects can effectively enhance the zincophilicity of graphene. Benefiting from the zincophilic heteroatom dopants, O-/N-doped graphene nanosheets significantly enhance Zn affinity at specific active sites, including ether oxygen (e1O), carboxylic oxygen (cO), and pyrrolic nitrogen sites (Fig. 4f) [68]. Furthermore, the introduction of a quadruple-defect configuration further increases the total Zn adsorption energy to −4.41 eV, which markedly enhances the zincophilicity compared to pristine Gr (Fig. 4g) [84]. Collectively, these chemical and structural modifications by defect and heteroatom doping substantially reduce the Zn nucleation barrier, thereby endowing the graphene substrate with the ability to induce uniform Zn nucleation.

Notably, the reversibility of Zn anodes depends on the coupled, countervailing electrochemical processes of deposition and stripping [87, 88]. A strong zincophilic nature implies strong bonding between Zn atoms and the substrate, which induces homogeneous nucleation and uniform Zn plating. However, for the stripping process, an excessively strong interaction between the substrate and the deposited Zn inhibits the removal of Zn atoms. Therefore, an appropriate level of zincophilicity is crucial for achieving highly reversible deposition/stripping processes. Otherwise, incomplete stripping will lead to irreversible Zn accumulation, dead Zn formation, and even electrode pulverization, ultimately deteriorating the electrodeposition reversibility. Inspired by the Sabatier principle in chemical catalysis, a Cu@Zn anode is designed [15]. The moderate interaction between Cu and Zn facilitates uniform Zn stripping throughout discharge and dendrite-free Zn plating throughout charge. Even under a high depth of discharge (DOD) of 80%, this anode delivers prolonged cycling stability exceeding 250 h.

## Lattice-Matched Design for Crystallographically Oriented Deposition

As elucidated in the previous chapter, zincophilicity modulation enables uniform initial Zn nucleation, which is the prerequisite for dendrite-free deposition. The subsequent growth stage determines the final morphology and crystallographic texture of the Zn deposit. Lattice matching engineering, the second core strategy of the multiscale regulation framework established in this review, achieves targeted regulation of the Zn growth stage via imposing geometric constraints through crystallographic coherence between the substrate and the growing Zn layer. This strategy induces ordered epitaxial deposition of Zn and fundamentally suppresses random dendrite formation during electrochemical plating. This chapter elucidates the mechanisms of lattice mismatch in regulating Zn epitaxial deposition, reviews recent lattice matching strategies for Zn anode stabilization, and analyzes their relationships.

### Effects of Lattice Mismatch on Zn Growth Behavior

**Fig. 5:**
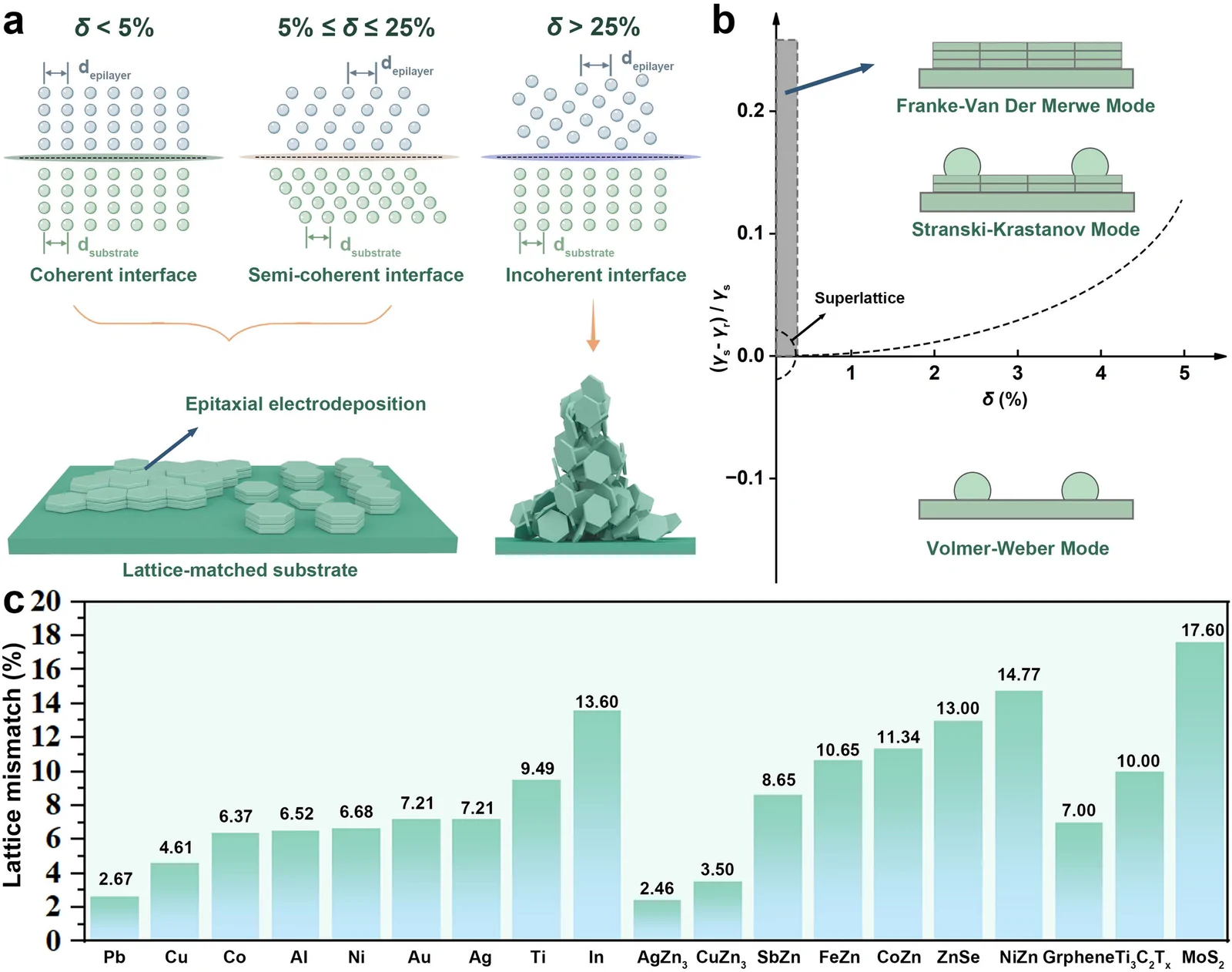
Influence of lattice mismatch on interfacial Zn growth. **a** Schematic illustrations of Zn epitaxial growth formed on substrates with coherent interfaces (*δ* < 5%), semi-coherent interfaces (5% ≤ *δ* ≤ 25%), and incoherent interfaces (*δ* > 25%). **b** Theoretical map delineating three nucleation regimes as a function of lattice mismatch (*x*-axis) and surface energy difference (*y*-axis), where γ_s_ and γ_f_ represent the surface energies of the substrate and the deposited film, respectively. Reproduced with permission [52]. Copyright 2024, Royal Society of Chemistry. **c** Lattice misfits between Zn and the common substrates. Reproduced with permission [41]. Copyright 2024, Wiley–VCH

The lattice mismatch (*δ*) between the Zn epilayer and the substrate is a critical parameter that determines the interfacial structure, the resultant Zn deposition morphology, as well as the microstructure and properties of the epilayer. This mismatch can be quantified and characterized by the difference in lattice constants, as described by Eq. (5) [48]:

5 $$\begin{array}{c}\delta = \frac{\left|{d}_{epi} - {d}_{sub}\right|}{{d}_{sub}}\end{array}$$

where ${d}_{epi}$ and ${d}_{sub}$ are the lattice parameters of Zn and substrate, respectively. As illustrated in Fig. 5a, based on the anticipated *δ* between the substrate and the epilayer, the contact interfaces can be categorized into three types: coherent, semi-coherent, and incoherent interfaces. At coherent interfaces (*δ* < 5%, e.g., commercial Zn foils), Zn nucleation tends to yield well-ordered crystalline deposits with relatively smooth interfaces [89]. For semi-coherent interfaces (5% ≤ *δ* ≤ 25%, e.g., graphene), the partial lattice matching creates crystallographic orientation locking, which strongly impacts the stability of the Zn deposition layer [90]. In contrast, at incoherent interfaces (*δ* > 25%, e.g., titanium foils), the large crystallographic disparity prevents effective epitaxial coupling [91]. The resulting high nucleation energy barrier favors preferential Zn^2+^ deposition on protruding nuclei, eventually causing uneven growth and zinc dendrite formation. Theoretically, the growth of deposit thin films generally follows three classical modes (Fig. 5b): Frank–Vander Merwe (layer-by-layer growth), Volmer–Weber (island growth), and Stranski–Krastanow (layer-plus-island growth) [52, 92]. These growth behaviors are principally governed by the *δ* between the deposits and the substrate. Ideally, to achieve the continuous, smooth Zn deposition layer dictated by the Frank–Vander Merwe growth model, the *δ* between the epilayer and the substrate must be minimized (see the area outlined by the dashed box in Fig. 5b). Practically, a sufficiently small *δ* enables the interface to maintain coherence; however, the accumulation of strain energy with increasing deposit thickness will eventually induce the formation of defects such as dislocations. Once *δ* exceeds a critical threshold, the strain energy surpasses the coherency limit of the interface, resulting in a loss of epitaxial registry. In this context, the critical thickness (*t*_c_) is defined as the maximum thickness at which defect-free epitaxial growth can be sustained, and it is intrinsically governed by the magnitude of lattice mismatch, as quantitatively described by Eq. (6) [93]:

6 $$\begin{array}{c}{t}_{c} = b{\left(\frac{{\delta}_{s}}{\delta }\right)}^{2}\end{array}$$

where* b* is the magnitude of the Burgers vector and $\delta_s$ represents the stability threshold parameter in one-dimensional epitaxial interface models. When the thickness of the epitaxial layer remains below this critical value, lattice mismatch can be accommodated elastically, allowing defect-free epitaxial growth. Conversely, once the thickness exceeds the critical threshold, stress relaxation becomes thermodynamically unavoidable, resulting in the generation of interfacial defects and disordered structures. Therefore, the fundamental elimination of lattice mismatch is critical to achieving ultra-stable, highly regular, and flat Zn epilayers. The structural characteristics of Zn epilayers are fundamentally governed by the substrate crystallographic anisotropy [94, 95]. As illustrated in Fig. 5c, systematic investigations have quantified the *δ* between Zn and various substrate materials, primarily encompassing metallic substrates (e.g., Pb, Cu, Co, Al, Ni, Au, Ag, Ti, and In) [52, 96], alloy materials (e.g., AgZn_3_, CuZn_5_, SbZn, FeZn, ZnSe, CoZn, and NiZn) [41, 97, 98], and two-dimensional materials (e.g., graphene, MXene, and MoS_2_) [90, 99, 100]. These comprehensive studies have established a fundamental database for the rational design of substrates that promote stabilized epitaxial deposition.

### Strategies for Interfacial Design with Low Lattice Mismatch

#### Single-crystal Zn Substrate Engineering

**Fig. 6:**
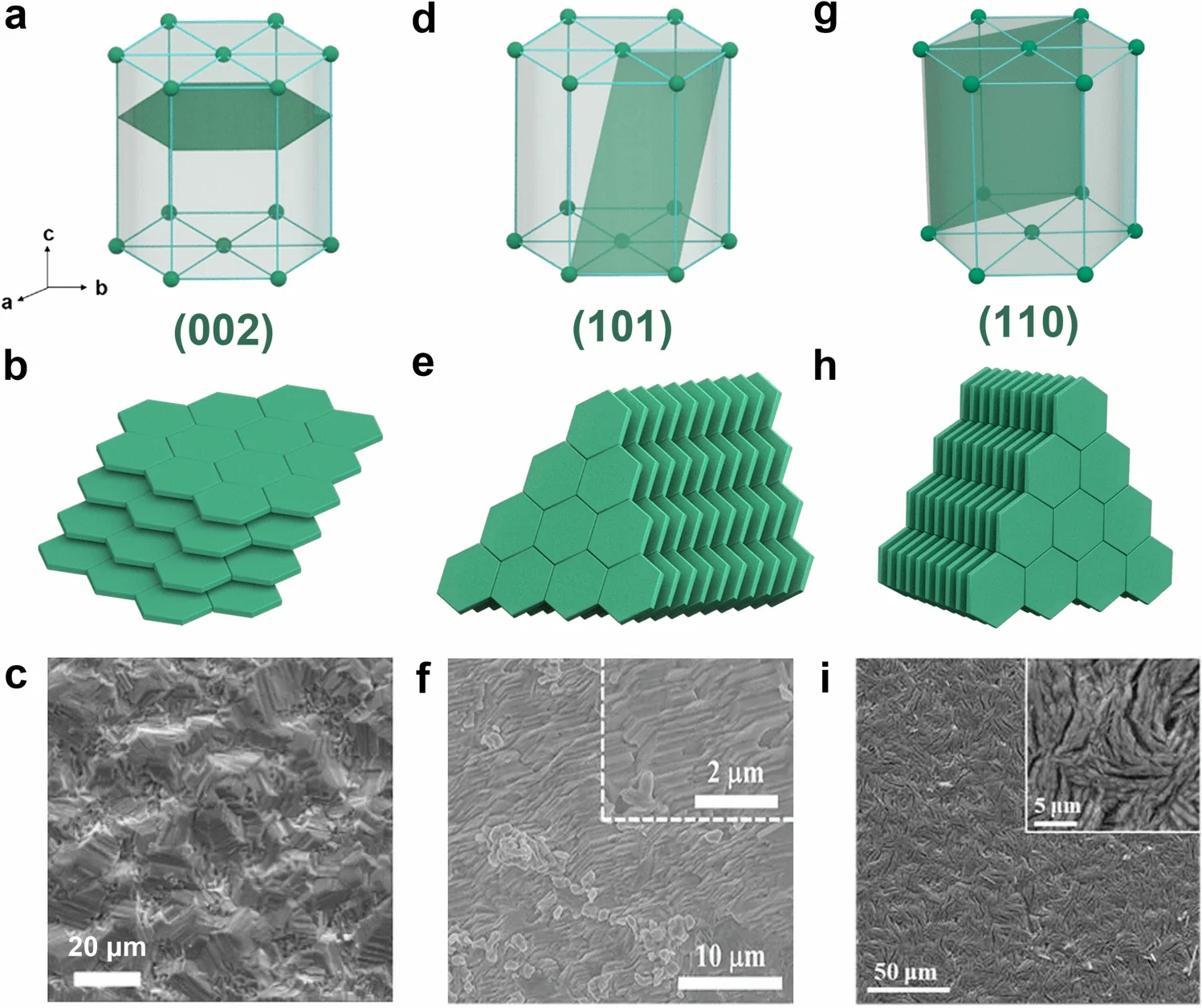
Epitaxial growth behavior on single-crystal Zn substrates with different crystallographic orientations. **a** Schematic illustration of the (002) lattice planes and **b** corresponding oriented deposition mode. **c** SEM image of the Zn(002) electrode surface after plating 0.67 mAh cm^−2^ in a symmetric Zn||Zn cell. Reproduced with permission [93]. Copyright 2024, Nature Publishing Group. **d** Schematic illustration of the (101) lattice planes and **e** corresponding oriented deposition mode. **f** SEM image of 3 mAh cm^−2^ Zn deposited on a (101)-textured Zn electrode. Reproduced with permission [109]. Copyright 2024, Wiley–VCH. **g** Schematic illustration of the (110) lattice planes and **h** corresponding oriented deposition mode. **i** SEM image of a Zn(110) electrode after 100 cycles in a symmetric Zn||Zn cell at a current density of 5.0 mA cm^−2^ with an areal capacity of 2.0 mAh cm^−2^. Reproduced with permission [110]. Copyright 2024, Nature Publishing Group

Precise lattice matching between the substrate and the Zn deposit ensures a uniform crystal orientation, thereby facilitating the fusion of individual electroplating islands into larger single crystals. However, the inherent polycrystalline and multi-oriented characteristics of commercial Zn substrates often result in random distribution of electrodeposition directions [101, 102]. Preferential growth of a single crystal plane is superior to the uncontrolled growth of multiple planes. Thus, constructing single-textured substrates enables lattice locking to steer the growth of specific favorable crystal planes, which is critical for regulating ordered Zn electrodeposition and enhancing anode stability. Metallic Zn crystallizes in a hexagonal close-packed (HCP) structure (space group P6_3_/mmc), and its electrochemical deposition behavior is primarily governed by four low-index crystal planes: (002), (100), (110), and (101) [103–105]. These facets exhibit distinct atomic arrangements, surface energies, and electrochemical activities, which lead to different Zn deposition behaviors and capabilities in mitigating parasitic side reactions [106, 107]. The (002)-textured Zn anodes exhibit low surface energy and weak electrochemical activity, which simultaneously suppress side reactions and promote horizontal hexagonal platelet growth (Fig. 6a, b) [108]. However, the inherent lattice mismatch at the epitaxial interface causes random and disordered growth of Zn when the deposition thickness exceeds the critical limit, ultimately leading to dendrite formation. To address this issue, the construction of [0001] uniaxially oriented Zn metal anodes with a single (002) texture enables layer-by-layer growth of Zn atoms following the “ ~ ABABAB ~ ” stacking sequence of closely packed (002) planes. This design fundamentally eliminates lattice mismatch at the epitaxial interface, achieves ultra-sustainable Zn homoepitaxial growth, and effectively suppress dendrite initiation and growth even under long-term cycling (Fig. 6c) [93]. The (100)-faceted Zn electrode enables vertically aligned deposit growth, which significantly suppresses corrosion and enhances deposition reversibility. The exceptional performance of (100) facet originates from its low electrochemical surface area, which is governed by surface diffusion barriers and deposition orientation [111]. Consequently, the (100)-oriented Zn electrodes develop thick, vertically aligned plates with minimal aspect ratios, uniformly oriented toward the separator, thereby effectively mitigating short-circuit risks. The highly (101) textured Zn anode sustains steady vertical epitaxial growth with improved mass transport, enabling persistent regulated deposition (Fig. 6d, e). Benefiting from directional lattice templating and strong interfacial adhesion, the anode facilitates sustained electroepitaxial growth, while the reduced grain boundary area effectively suppresses side reactions even under high areal capacity conditions (Fig. 6f). The (101)-textured Zn symmetric cells exhibited remarkable stability across current densities from 1 to 40 mA cm^−2^, achieving an ultrahigh cumulative plating capacity of 10.7 Ah cm^−2^ [109]. The (110)-textured Zn electrode also exhibits vertically aligned deposits. In contrast to the (100) facet, the Zn platelets grown on the (110) crystal facet possess a larger lateral size (Fig. 6g, h). A coherent interface is formed between the Zn(110) substrate and the later deposited Zn, where epitaxial Zn tightly adheres to the substrate without observable lattice distortions or dislocations. Consequently, the uniaxially oriented Zn(110) anode enables spontaneous intrinsic, dislocation-free epitaxial growth, resulting in highly uniform Zn deposition (Fig. 6i) [110].

Nevertheless, single-crystal Zn anodes have inherent drawbacks. Repeated Zn plating/stripping induces large volume change and stress, causing recrystallization, grain boundary formation, and crystal plane transformation. The single-crystal structure gradually degrades into polycrystalline, losing its original orientation advantages. Meanwhile, aqueous corrosion generates by-products that passivate the interface and disturb uniform Zn deposition. In addition, the stringent preparation conditions and high production cost also hinder the large-scale fabrication of defect-free single-crystal Zn foils for practical industrial applications.

#### Low Lattice Mismatch Substrate Engineering

**Fig. 7:**
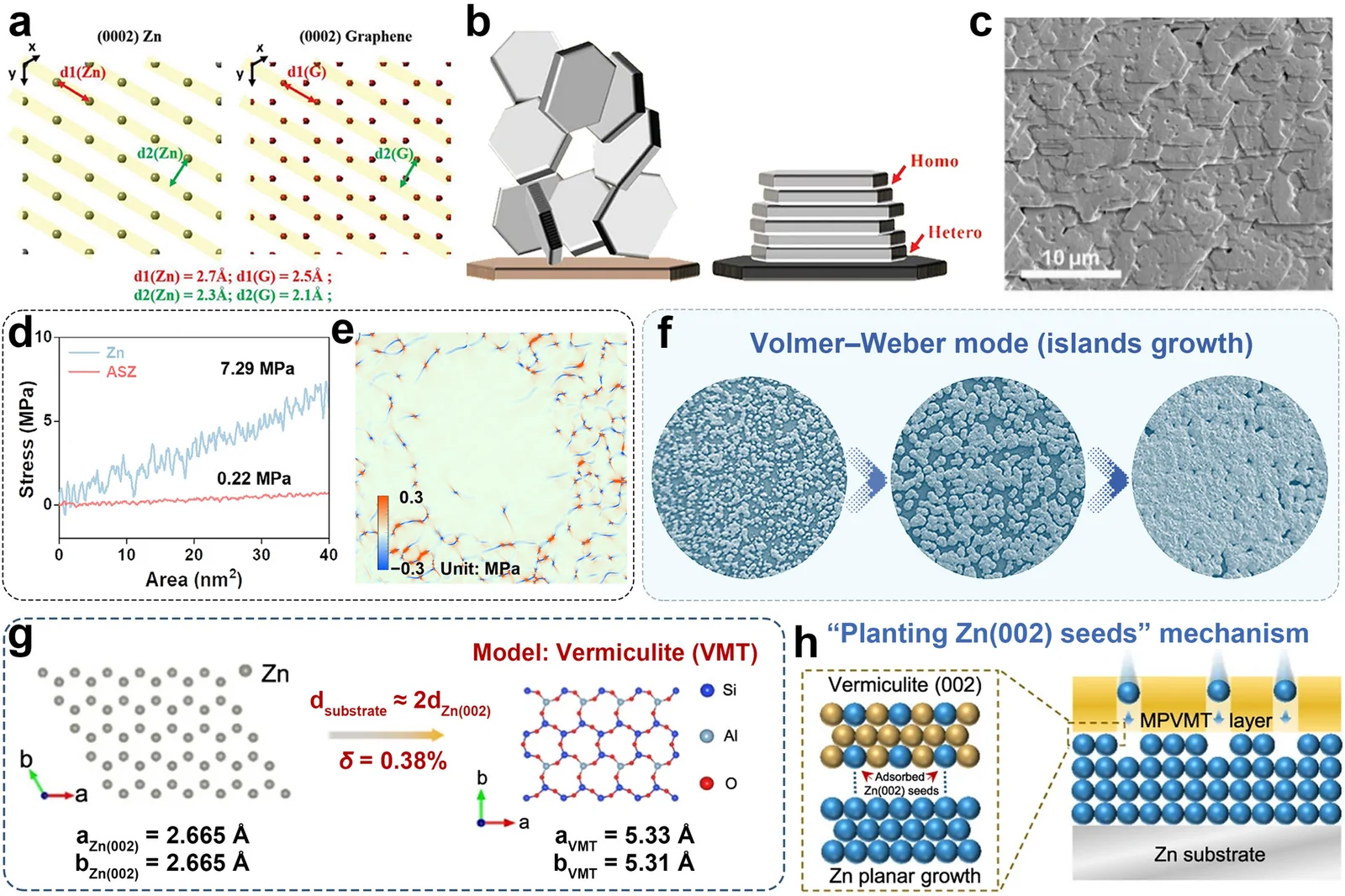
Interfacial Zn growth behavior on substrates with low lattice mismatch. **a** Schematic illustration of the lattice mismatch between graphene(0002) and the Zn(002) plane. **b** Design principle for epitaxial Zn electrodeposition. Reproduced with permission [90]. Copyright 2019, American Association for the Advancement of Science. **c** SEM image of electrodeposited Zn on Cu(111) at 10 mA cm^−2^ with an areal capacity of 1 mAh cm^−2^. Reproduced with permission [114]. Copyright 2025, American Chemical Society. **d** Analysis of lattice strain accumulation during Zn deposition. **e** Strain measurements of the deposited Zn layer on an Al-0.1wt%Si substrate. Reproduced with permission [115]. Copyright 2025, Wiley–VCH. **f** Schematic illustration of Zn nucleation and growth on the ZnCO_3_ autogenous regulating layer. Reproduced with permission [116]. Copyright 2025, Wiley–VCH. **g** Schematic illustration of design of MPVMT layers based on the extended substrate screening strategy for stabilizing Zn anodes. **h** Schematic illustration of nucleation behavior on the MPVMT layer. Reproduced with permission [117]. Copyright 2024, Nature Publishing Group

Constructing low-lattice-mismatch heteroepitaxial interfaces is another effective strategy to regulate Zn deposition behavior. A minimal lattice mismatch between the anode substrate and the Zn(002) plane lowers the interfacial energy, thereby thermodynamically favoring the nucleation and growth of highly (002)-textured Zn [112, 113]. Since Archer’s team pioneered oriented Zn(002) deposition via a graphene interface in 2019, epitaxial Zn deposition strategies based on low lattice mismatch substrate engineering have advanced considerably [90]. Two-dimensional crystalline materials with long-range ordered lattice structures have been widely explored for Zn anode modification, because of their adjustable lattice parameters and minimal lattice mismatch with Zn. A highly oriented graphene coating was fabricated on the stainless steel surface via a fluid-mediated shear alignment technique, forming an intrinsic semi-coherent interface with a 7% lattice mismatch between the graphene(0002) and Zn(002) planes. This interface facilitates strain relaxation and promotes epitaxial growth (Fig. 7a). The subsequent Zn deposition proceeds from heteroepitaxial nucleation to lateral homoepitaxial expansion, forming uniform textured Zn layers (Fig. 7b). This interfacial oriented deposition behavior remarkably optimizes the electrochemical performance and cycling durability of the Zn anode, further uncovering the critical mechanism by which graphene modification regulates the electrochemical behavior of AZIBs. Metallic and alloy substrates featuring low lattice mismatch with Zn and excellent electrical conductivity have also been extensively investigated for Zn anode design. Copper (Cu), with its face-centered cubic (fcc) close-packed structure, has been widely studied as a template for orienting Zn(002) deposition due to its excellent lattice matching [118, 119]. Xiao et al. further validated the epitaxial relationship between Cu(111) and Zn(002) through pole figure analysis, revealing a 60° in-plane orientation alignment and a minimal facet misfit of 2.3%. This near-ideal lattice coherence enabled highly ordered Zn deposition, delivering a Coulombic efficiency of 99.93%, an areal capacity of 20 mAh cm^−2^, with dendrite-free morphology over extended areas (Fig. 7c) [114]. Ultralow lattice mismatch at the Zn/substrate interface enables precise crystallographic modulation of oriented Zn deposition, which drastically mitigates intergranular strain and enables long-term cycling of AZIBs [120]. An Al-Si alloy substrate with an ultralow lattice mismatch of 0.51% was developed to enable sustained (002)-plane epitaxial Zn growth [115]. Quantification of lattice distortion revealed a much lower stress value for the electrodeposited Zn layer on this alloy (0.22 MPa) than for that on pristine Zn (7.29 MPa), indicating a compact lattice arrangement under oriented growth (Fig. 7d). The corresponding strain map further revealed a significantly lower and more uniform stress field (−0.3 to 0.3 MPa) in comparison with pristine Zn substrates (Fig. 7e), demonstrating that the Al-0.1wt% Si substrate effectively mitigates stress concentration and diminishes the driving force for dendrite formation. The Al-Si-Zn||Cu half cells achieve a Coulombic efficiency of 99.83%, whereas the symmetric Al-Si-Zn cells cycle stably for 8000 h at 5 mA cm^−2^. This superior performance stems from the negligible lattice spacing discrepancy between Al-0.1wt% Si(111) and Zn(002), which facilitates atomically flat Zn nucleation and suppresses hierarchical twin formation, thereby realizing a near-zero-strain Zn anode [115]. In situ construction of intrinsic crystalline interlayers with low lattice mismatch on the Zn matrix surface is another emerging strategy to regulate Zn epitaxial growth [121, 122]. A weakly conductive, oriented ZnCO_3_ layer can be generated directly on the Zn matrix surface through a facile autogenous reaction, which features an extremely small lattice mismatch of only 9.72% between its (1121) crystal plane and Zn(002). Leveraging this structural advantage, the autogenous ZnCO_3_ layer enables a Volmer–Weber Zn deposition mechanism, ultimately yielding a dense and dendrite-free morphology (Fig. 7f) [116]. Traditional substrate screening relies on the 1:1 lattice parameter matching criterion (d_substrate_: d_Zn(002)_ = 1:1), under which coherent interfaces can only be formed by single-crystal Zn(002) anodes and a few Zn-based alloys [123]. To break this limitation, Zheng et al. proposed the extended substrate screening strategy, selecting vermiculite (lowest lattice mismatch of 0.38%, d_substrate_ ≈ 2d_Zn(002)_) as the model and fabricating a large-scale, green monolayer porous vermiculite (MPVMT) as a Zn electrode functional coating (Fig. 7g) [117]. The unique “planting Zn(002) seeds” mechanism drives Zn adatoms to preferentially occupy the vermiculite lattice’s hollow sites, which guides the subsequent horizontal growth of Zn and achieves a dendrite-free Zn electrode (Fig. 7h). Moreover, the surface hydrophilic groups on the non-conductive MPVMT effectively suppresses the HER and by-product formation, thereby enabling excellent cycling stability at high current densities.

However, even low-mismatch substrates (e.g., graphene, Cu(111)) present inherent lattice differences with Zn. Long-term cycling induces interfacial stress, dislocations, and stacking faults, which weaken the epitaxial effect and disrupt oriented deposition, eventually triggering dendrite growth and side reactions. Furthermore, the epitaxial effect is usually effective for thin-layer Zn deposition. Under high Zn loading conditions required for practical applications, severe strain accumulation and defect proliferation will occur, leading to the rapid failure of epitaxial growth control.

## 3D Structural Engineering for Homogenized Zn Deposition

As established in the previous chapters, zincophilicity regulation lowers the nucleation barrier and lattice matching achieves interfacial control of Zn epitaxial deposition, the spatial evolution of Zn deposits during growth is also governed by electric field distribution and ion flux. 3D structural engineering realizes microscale spatial confinement of the full Zn deposition process via tailoring the topological structure of Zn anode matrix. This strategy homogenizes local current density, regulates ion transport pathways, and provides physical confinement for deposited Zn, thereby preventing uncontrolled dendritic propagation. This chapter will elucidate the functional mechanisms, summarize the structural optimization strategies for 3D Zn anode matrices, and analyze their structure-property relationships in practical electrochemical operation.

### Functional Roles of 3D Architectures

**Fig. 8:**
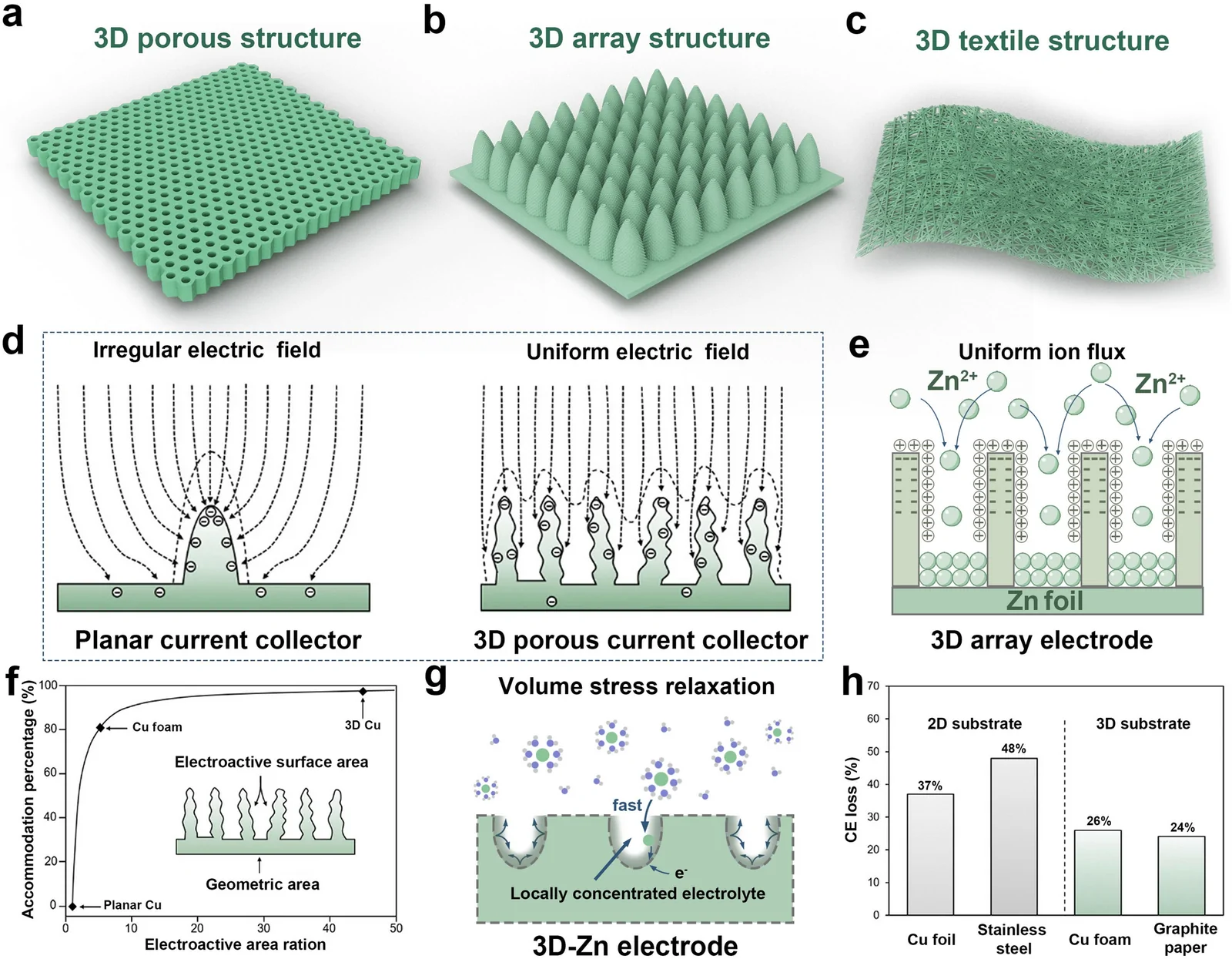
Functional roles of 3D substrates in regulating Zn deposition. Schematic illustrations of electrodes with **a** a 3D porous structure, **b** a 3D array structure, and **c** a textile structure. **d** Simulated electric field distribution on planar and 3D substrates during Zn deposition. Reproduced with permission [52]. Copyright 2024, Royal Society of Chemistry. **e** Schematic depiction of uniform ion flux distribution enabled by a 3D array electrode during Zn deposition. **f** Correlation between the volume accommodation percentage and the electroactive area ratio in 3D substrates. Reproduced with permission [132]. Copyright 2015, Nature Publishing Group. **g** Schematic illustration of volumetric stress mitigation in 3D Zn electrodes. Reproduced with permission [138]. Copyright 2021, Elsevier. **h** Influence of calendar aging on the Coulombic efficiency of Zn metal anodes with different substrate architectures. Reproduced with permission [140]. Copyright 2023, American Chemical Society

At the microscopic scale, the transition from planar to 3D geometries represents a pivotal shift in managing the spatiotemporal evolution of Zn deposition. Various 3D substrates, including porous structures (Fig. 8a), arrayed architectures (Fig. 8b), and textile configurations (Fig. 8c), have been successfully applied to Zn anodes [124–126]. The topological structure directly regulates the electric field and/or ion concentration distribution, which in turn governs the nucleation and growth behavior during Zn electrodeposition [127]. In conventional 2D metal anodes (e.g., Zn foil), even negligible surface protrusions trigger a self-amplifying “tip effect” by localizing the electric field, which accelerates Zn accumulation at these sites and ultimately leads to dendrite formation and separator penetration. In contrast, the large specific surface area of 3D substrates intrinsically lowers the local current density across the electrode scaffold and facilitates a uniform electric field distribution within the internal space of the framework [128]. As a result, metallic Zn preferentially nucleates and grows within the internal space of the framework instead of the external surface, which effectively suppresses dendrite generation (Fig. 8d). The interconnected internal pores of the 3D architecture also provide sufficient diffusion pathways for Zn^2+^ ions, which optimizes ion transport kinetics, reduces interfacial concentration gradients, and thus achieves uniform ion flux across the entire electrode (Fig. 8e) [129–131]. Meanwhile, the 3D substrate provides a large electrochemically active surface area, enabling high areal capacity operation without inducing localized deposition (Fig. 8f) [132–134].

The 3D architecture also addresses the volume variation issue that plagues 2D planar Zn anodes. Uneven Zn deposition on 2D electrodes forms dendritic, mossy, or rod-like morphologies. Such undesirable deposition patterns remarkably increase volume variation, induce the formation of dead Zn, and even cause separator piercing and internal short circuits. The sufficient internal void space of the 3D matrix accommodates the volume change of deposited Zn, effectively relieves internal stress induced by metal deposition, and keeps the electrode structurally stable during long-term cycling (Fig. 8g) [135–137]. Furthermore, 3D substrates with precisely tunable nanopores enable the formation of concentrated electrolytes localized at the interface [138]. Nanopore space-charge effects tailor ion distribution in the electric double layer (EDL), forming concentrated interfacial electrolyte with no impact on the bulk [52, 139]. Owing to their unique EDL characteristics (fewer free water molecules and abundant available Zn^2+^), concentrated electrolytes are recognized as highly capable of protecting Zn anodes. Additionally, 3D electrodes can mitigate loss of capacity during the calendar aging of Zn anodes (Fig. 8h) [140]. A possible explanation for this performance improvement is the enlarged specific surface area of 3D substrates. The 3D structure can provide continuous mass transfer pathways and maintain favorable interfacial reaction kinetics, which helps suppress the generation of inactive byproducts and improve long-term electrochemical stability.

### Strategies for 3D Structural Construction

#### Structural Optimization of 3D Substrates

**Fig. 9:**
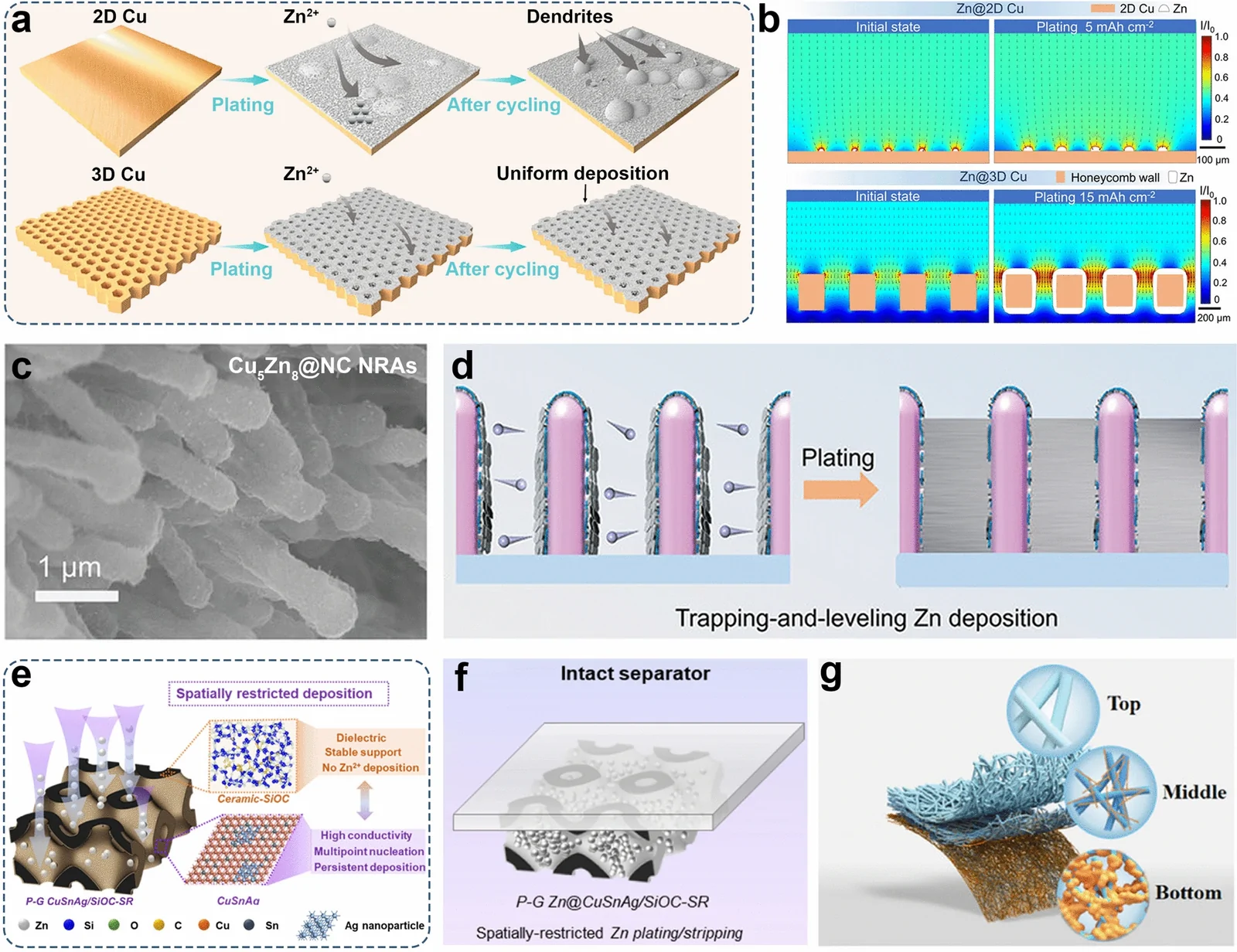
Design of 3D substrates for regulating Zn deposition behavior. **a** Schematic illustration depicting the morphological evolution of Zn anodes employing different substrate architectures after plating and prolonged cycling. **b** Simulated relative intensity distribution of the local electric field for Zn@2D Cu and Zn@3D Cu electrodes. Reproduced with permission [141]. Copyright 2026, Wiley–VCH. **c** SEM image showing the synthesis of Cu_5_Zn_8_@NC nanorod arrays. **d** Schematic illustration of the trapping-and-leveling mechanism for Zn deposition on Cu_5_Zn_8_@NC nanorod arrays. Reproduced with permission [142]. Copyright 2024, Wiley–VCH. **e** Schematic of Zn deposition behavior on CuSnAg/SiOC electrodes. **f** Zn deposition state of P-G Zn@CuSnAg/SiOC-SR electrode. Reproduced with permission [143]. Copyright 2024, Elsevier. **g** Schematic illustration of the gradient architecture design of DG-Zn electrodes. Reproduced with permission [144]. Copyright 2025, Wiley–VCH

The structural characteristics of 3D Zn anode matrices fundamentally dictate their Zn deposition regulation via homogenizing the electric and/or ionic concentration distributions around the substrates. A highly precise honeycomb-patterned Cu substrate with controllable pore dimensions is engineered to facilitate uniform Zn electrodeposition and effectively suppress Zn dendrite growth (Fig. 9a). Compared with planar 2D Cu current collectors, the 3D honeycomb Cu substrate reduces the local current density and optimizes the electric field distribution across the electrode, enabling homogeneous Zn deposition (Fig. 9b) [141]. Despite the performance improvements brought by conventional 3D frameworks, Zn deposition still tends to occur preferentially on the upper surface of the architecture rather than in the internal voids, especially under high current densities and large areal capacities. This uncontrolled top-growth behavior causes insufficient utilization of internal space and eventually leads to dendrite formation on the electrode surface. Targeted structural optimization strategies have been developed to address this critical issue, realizing confined and uniform Zn deposition within the 3D framework. A core-shell nanorod array with a hierarchical topology consisting of N-doped carbon layers embedded with the zincophilic Cu_5_Zn_8_ alloy is fabricated to stabilize Zn anodes (Fig. 9c). The hierarchical nanorod arrays guide inward Zn^2+^ flux through confined transport channels, and the interior Cu_5_Zn_8_ zincophilic sites induce preferential inside-out nucleation. This design homogenizes local ion flux and effectively suppresses top-preferred deposition and tip-induced dendrite growth (Fig. 9d). Thus, the optimized electrode shows a Coulombic efficiency of 99.83% throughout 5000 plating/stripping cycles, and the symmetric cell enables an ultra-durable dendrite-free cycling of 7000 h, accompanied by a low overpotential of 16.5 mV at 1 mA cm^−2^/1 mAh cm^−2^ [142]. A localized-activation design principle has also been proposed for 3D Zn anodes to overcome the uncontrolled “top-growth” dendrite issue. Based on this principle, a spatially confined 3D Zn@CuSnAg/SiOC anode has been constructed, where the top surface adjacent to the separator is modified with an insulating SiOC layer, and the internal surfaces are functionalized with highly reactive Zn@CuSnAg composite (Fig. 9e) [143]. This unique spatial design inhibits Zn plating/stripping at the upper side, and enables localized Zn deposition inside the 3D framework, which eliminates top-growth Zn dendrites and maximizes the utilization of the internal void space (Fig. 9f). Gradient structural design is an alternative approach for regulating the spatial distribution of Zn deposition. Li et al. successfully fabricated a flexible 3D nanofibrous Zn anode combining dual gradients of electrical conductivity and pore size [144]. The nanofibrous membrane displays a continuous gradient distribution along the thickness direction, with pore size gradually decreasing and electrical conductivity progressively increasing from the top down. The vertical conductivity gradient drives preferential Zn plating at the anode base, whereas the porosity gradient ensures rapid Zn^2+^ transport and maintains sufficient ion supply in the internal space. The dual-gradient and 3D structural synergy induces a Zn plating regime from the bottom upward, providing effective dendrite mitigation. In addition, the 3D structure relieves the internal volume expansion of the anode, significantly mitigating structural deformation during Zn electrodeposition and ensuring negligible variation in anode thickness before and after plating.

#### Functional Modulation of 3D Substrates

**Fig. 10:**
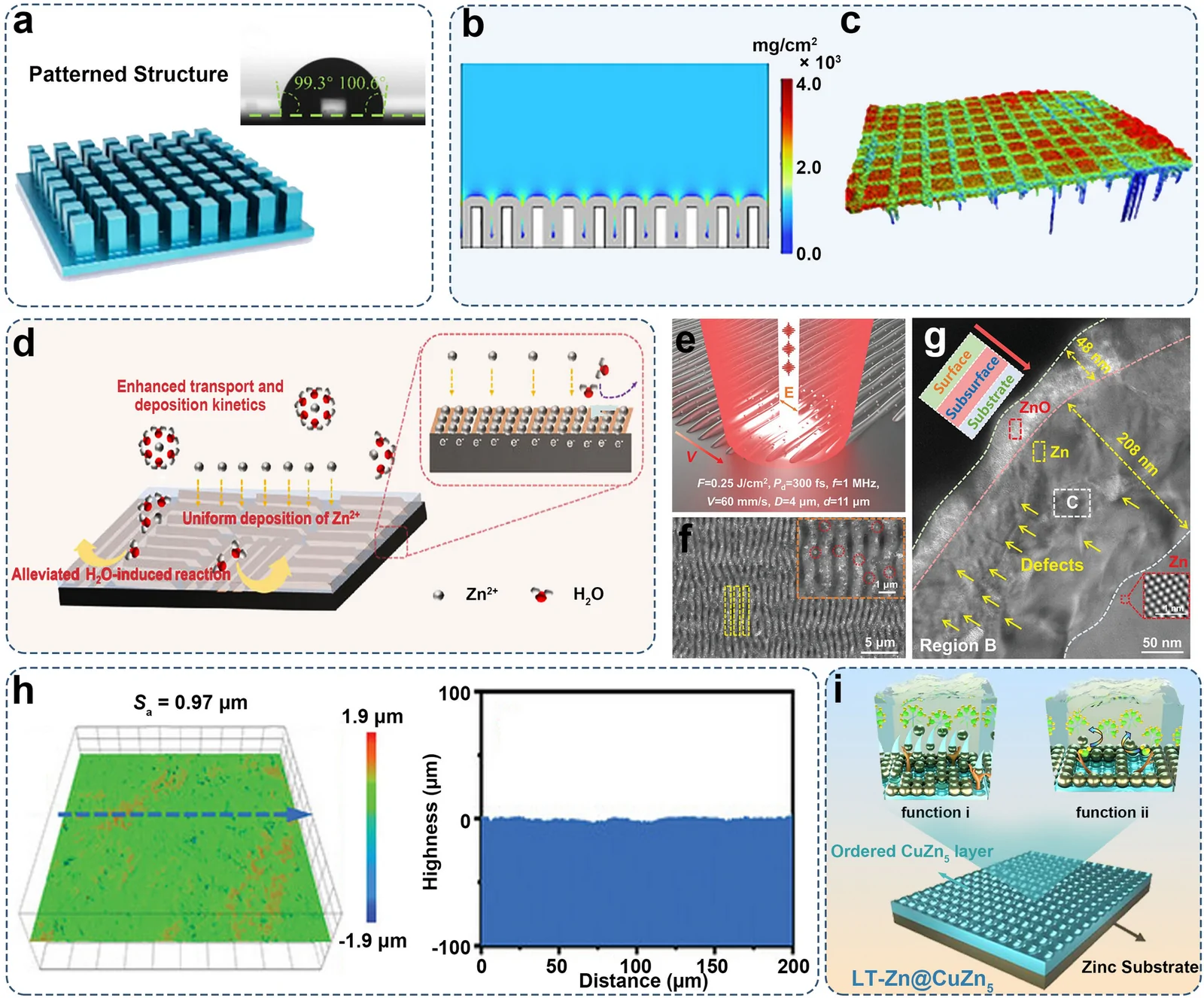
Intrinsic 3D substrate interface engineering for inhibiting side reactions. **a** Schematic illustration of the micro-hydrophobic micro/nanostructure. **b** COMSOL simulation of Zn^2+^ deposition on the Zn100° electrode surface in a Zn||Zn symmetric cell at 40 s. **c** 3D morphology after cycling at 2 A g^−1^ of 2000 cycles. Reproduced with permission [147]. Copyright 2026, Wiley–VCH. **d** Schematic illustration of Zn deposition on the LZ@Zn anodes. Reproduced with permission [148]. Copyright 2025, Wiley–VCH. **e** Schematic of the femtosecond laser processing strategy for fabricating LIPSS on Zn metal. **f** SEM image of LIPSS (yellow rectangle and red circle highlight the micro grooves and nanoparticles, respectively). **g** HRTEM image of LZ@Zn anodes. **h** 3D image of Zn-LIPSS anode deposited at 5 mA cm^−2^/9 mAh cm^−2^. Reproduced with permission [149]. Copyright 2025, Wiley–VCH. **i** Schematic illustrating the function of LTZn@CuZn_5_ in regulating Zn electrodeposition. Reproduced with permission [150]. Copyright 2026, American Chemical Society

While 3D substrate engineering effectively mitigates dendrite growth by modulating the electric field and/or ion concentration distribution, it fails to fundamentally eliminate the parasitic side reactions induced by the weakly acidic aqueous electrolyte (e.g., HER and corrosion). Critically, the high specific surface area introduced by 3D architectures exposes more electrochemically active sites, which in turn accelerates these detrimental interfacial side reactions. To address this limitation, functional modulation of 3D substrates, which is realized by in-situ constructing 3D interconnected networks via chemical or physical routes with intimate bonding and no external prefabricated materials, offers a solution to interfacial challenges. Unlike conventional ex-situ coatings that suffer from weak physical adhesion and inevitable inactive components, these in situ formed interconnected networks benefit from intrinsic integration with the Zn matrix, effectively isolating active Zn from corrosive electrolytes while avoiding interfacial adhesion failure and additional inactive phases [86, 145, 146]. The wettability regulation of 3D micro/nanostructures presents an inherent trade-off. Enhanced hydrophilicity improves electrolyte wettability, promotes Zn^2+^ migration and suppresses dendrite formation, but it significantly increases the solid-liquid reaction interface area, inevitably exacerbating HER and corrosion. Conversely, excessive hydrophobicity impedes adequate electrolyte access to the Zn anode, resulting in sluggish ion transport kinetics and drastically increased interfacial polarization, which ultimately degrades electrochemical performance. Accordingly, achieving a synergistic balance between wettability and electric field distribution is essential in 3D micro/nanostructure design. A micro-hydrophobic array with a contact angle of 100° was constructed by adjusting the geometric parameters of the microarray, thereby balancing ion transport kinetics and side reaction suppression (Fig. 10a) [147]. The moderate micro-hydrophobicity effectively reduces the exposure of active Zn to free water molecules, significantly lowering HER and corrosion rates. Thus, Zn deposition occurs homogeneously over the entire electrode surface, forming a flat and dense deposit. After 2000 full-cell cycles, the micropillars retain their structural integrity (Fig. 10b, c).

Although hydrophobic interphases effectively suppress water-induced parasitic reactions, they inevitably lead to deteriorated ionic conductivity and sluggish Zn deposition kinetics. To overcome this inherent bottleneck, the rational integration of 3D architecture with tailored interfacial chemistry represents a viable strategy. A 3D fish-scale-like lamellar architecture with zincophilic and hydrophobic properties was fabricated in-situ via etching and complexation to mitigate parasitic side reactions [148]. This architecture suppresses water-induced side reactions by repelling free water molecules, and simultaneously enhances Zn deposition kinetics, thereby enabling uniform Zn deposition (Fig. 10d). Furthermore, a periodic micro/nanostructure consisting of a surface oxide layer and a subsurface dislocation layer was fabricated by laser processing (Fig. 10e, g). This 3D architecture promotes a uniform Zn^2+^ flux. Meanwhile, the ZnO layer inhibits parasitic reactions and promotes Zn^2+^ transport, while the underlying dislocation layer provides electron pathways and mechanical buffering [149]. Consequently, the anode maintains stable and smooth Zn deposition (Fig. 10h). Alloying modification improves the anti-corrosion performance of 3D micro/nanostructures by forming a stable alloy phase that isolates the substrate from corrosive species. A laser-assisted alloying technique was employed to in situ generate CuZn_5_ alloy phases on 3D microstructured Zn surfaces (Fig. 10i) [150]. The as-optimized electrode features a geometrically ordered surface topology that homogenizes the local electric field, while the in-situ-formed CuZn_5_ alloy layer effectively suppresses hydrogen evolution. Given these synergistic effects, the electrode shows markedly enhanced electrochemical reversibility during Zn plating/stripping. It delivers stable cycling for over 3000 h at 3 mA cm^−2^ and 1 mAh cm⁻^2^, whereas pristine Zn short circuits after only 33 h.

3D structured electrodes homogenize local current density and buffer volume fluctuations over repeated Zn plating-stripping cycles. However, they inevitably introduce a high proportion of inactive scaffold materials and increased internal porosity, drastically reducing the full cell’s energy density. Additionally, the complex fabrication also increases manufacturing difficulty and cost. More importantly, these macroscopic structural designs alone provide insufficient control over the microscopic nucleation and crystallographic growth of Zn. Most scaffold materials exhibit intrinsically weak zincophilicity, leading to sparse nucleation sites with high nucleation overpotentials. Meanwhile, the lack of lattice matching between the scaffold and Zn results in randomly oriented crystal growth, which readily evolves into dendrites during long-term cycling. Therefore, a multiscale synergistic design strategy is required to achieve an electrode that simultaneously integrates interfacial chemistry regulation, crystallographic orientation control, and macroscopic current/ion distribution.

## Conclusion and Perspective

### Conclusions

In summary, the large-scale commercialization of AZIBs is severely impeded by uncontrollable dendrite growth and parasitic interfacial reactions at the Zn metal anode, which critically deteriorate Coulombic efficiency and cycle life. Targeted modulation of the nucleation and growth stages across the entire Zn deposition process can effectively guide uniform nucleation and compact growth, offering a fundamental solution to Zn anode instability. This review systematically deconstructs the five sequential stages of Zn electrochemical deposition, clarifies the origins of deposition inhomogeneity and anode failure, and defines the core regulatory targets for Zn anode design. Building on this mechanistic foundation, we propose a full-chain multiscale regulatory framework from the atomic, interfacial and microscale structural characteristics of the Zn matrix for Zn anode modification, which integrates three core complementary strategies: zincophilicity regulation, lattice matching engineering, and 3D structural engineering. For each strategy, we provide a detailed analysis of the underlying mechanisms, design principles, and structure-property relationships, coupled with the latest advancements in the field. This approach clarifies the cross-scale design principles and emphasizes the shift from trial-and-error methods to rational material design. In conclusion, this review emphasizes the synergistic regulation of the entire Zn nucleation-growth process, establishing key structure-property relationships and design criteria for high-stability, dendrite-free Zn metal anodes. These insights offer invaluable guidance for advancing AZIB performance and its commercial feasibility.

### Perspectives

**Fig. 11:**
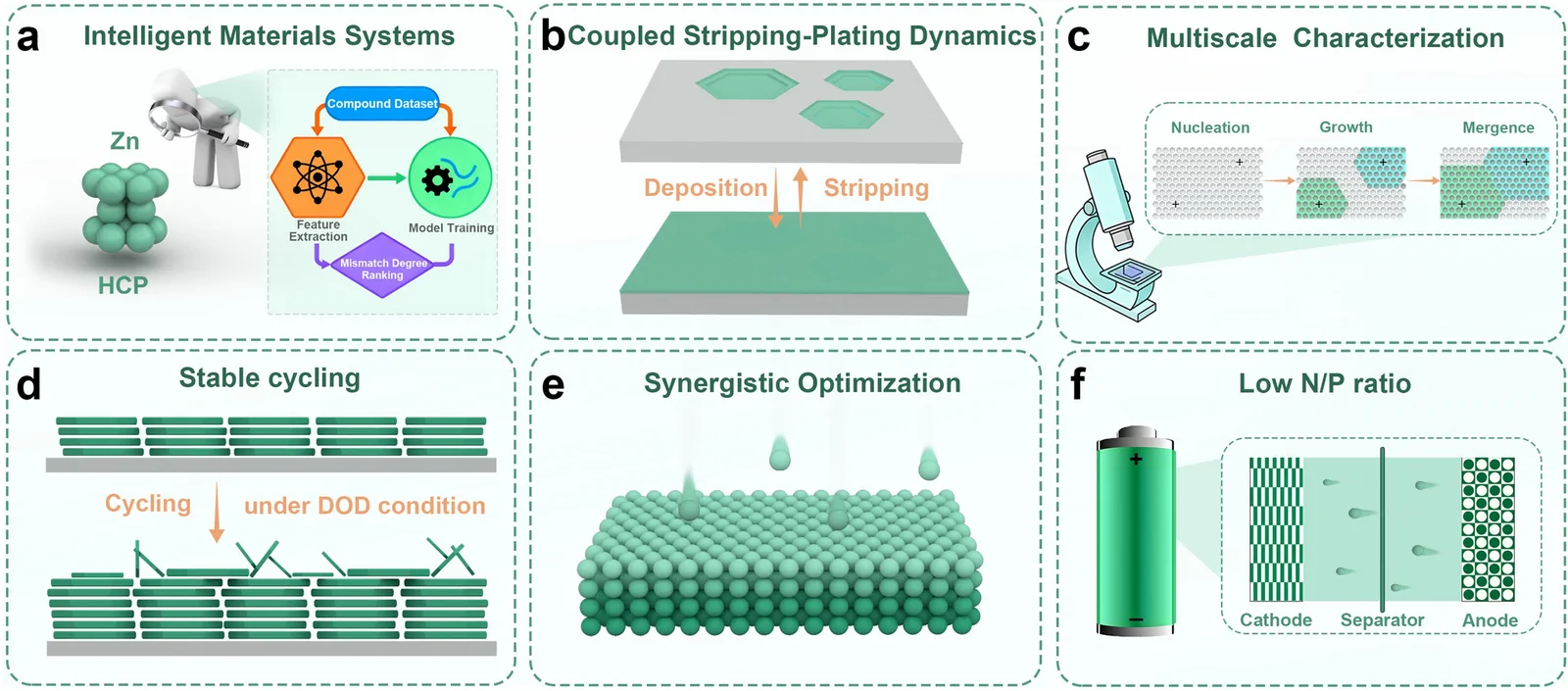
Prospects for AZIBs based on Zn anode design. **a** Schematic illustration of machine learning-assisted rational design for Zn anodes. **b** Regulation of coupled Zn stripping–plating dynamics processes. **c** Multiscale operando characterization toward mechanistic understanding. **d** Multiscale Zn anode engineering for long-term stable cycling. **e** Synergistic integration of Zn anodes with other battery components. **f** Scalable fabrication of Zn anodes for high Zn utilization

Zn anode design has emerged as a transformative approach to stabilizing Zn anodes in AZIBs, thereby effectively suppressing dendrite growth and enhancing electrochemical reversibility. Notwithstanding substantial progress in this field, numerous challenges persist that impede the practical implementation. Addressing these critical bottlenecks necessitates a deeper, multiscale understanding of Zn deposition mechanisms and their direct correlation with battery performance. Future research should prioritize the following interconnected directions (Fig. 11):

i. *Machine learning-assisted rational design of Zn anodes*: Machine learning (ML) and high-throughput computations open new avenues for the multiscale synergistic design of Zn anodes (Fig. 11a). By establishing quantitative correlations between key parameters including zincophilicity, surface energy, lattice mismatch, structural topology and electrochemical stability, high-efficiency screening of candidate materials can be realized, which transcends the limitations of traditional trial-and-error approaches [78, 151, 152]. Relevant descriptor systems are classified according to different design strategies: zincophilicity regulation relies on binding energy, nucleation energy barrier and nucleation overpotential; lattice matching design adopts lattice mismatch, interfacial strain, critical thickness and preferred orientation consistency as evaluation indicators; 3D structural engineering employs quantifiable metrics including specific surface area, electroactive area ratio, pore size and porosity, local current density distribution, Zn^2+^ flux homogeneity and volume accommodation capability. This synergistic integration of ML-guided discovery with rational design principles for Zn anodes will establish a complete rational design system for Zn anodes toward achieving highly reversible, dendrite-free Zn metal anodes.
ii. *Regulation of coupled stripping-plating dynamics processes*: Zn stripping is far from a simple reversal of deposition. A perfectly uniform deposit cannot ensure complete reversibility of stripping, as non-uniform dissolution disrupts crystallographic continuity, generates surface defects, and ultimately promotes random nucleation in subsequent cycles. Therefore, future research should move past the sole pursuit of dendrite‑free deposition and instead aim for topological stability, in which the Zn anode retains its structural and morphological integrity throughout repeated plating and stripping. For example, the construction of dynamically adaptive interphases can maintain conformal protection during the volumetric contraction inherent to the stripping phase. Zn anode design should simultaneously regulate the symmetry of Zn plating and stripping processes, realizing fully reversible electrochemical cycling (Fig. 11b).
iii. *Multiscale operando characterization for mechanistic elucidation*: Under complex electrochemical environments, the underlying mechanisms of existing regulation strategies remain incompletely elucidated, underscoring the necessity for in-depth investigation using multiscale in situ characterization techniques [153]. In situ microscopy and synchrotron-based methods can directly track morphology changes throughout cycling. Meanwhile, operando spectroscopy elucidates the interfacial chemistry and ion-transport dynamics during repeated charge and discharge [154, 155]. These theoretical insights guide the rational design of high-performance Zn anodes. Future research could combine specific in-situ techniques with electrochemical phenomena. For example, integrating in situ AFM with Cryo-TEM can reveal the nanomechanical properties of the protective interface and the atomic arrangement of nucleation seeds, without beam-induced artifacts. Additionally, coupling EQCM with in situ MRI can separate parasitic reaction mass from active Zn deposition and visualize concentration gradients within the porous structure. These advances in operando techniques will connect local interfacial interactions to overall battery degradation (Fig. 11c).
iv. *Multiscale Zn anode design for stable cycling*: Under high current densities and DOD, accelerated dendrite growth and side reactions, along with volumetric changes and repeated Zn stripping/plating, severely compromise electrode structural integrity. The resulting cracks and voids disrupt charge transfer uniformity, drastically reducing Zn deposition/stripping reversibility (Fig. 11d). Conventional single-scale designs (e.g., solely optimizing zincophilicity or 3D structures) cannot simultaneously ensure uniform deposition, suppress side reactions, and maintain structural stability [156]. Notably, zincophilicity regulation, lattice matching design and 3D structural engineering are not completely independent. In most functional modification strategies, these effects often interact and overlap with one another. For example, many zincophilic substrates can simultaneously expose well-matched crystal facets to achieve lattice coherence with Zn deposits; 3D gradient zincophilic structures can achieve bottom-to-top homogeneous and compact Zn deposition even under high DOD conditions. Therefore, a multiscale synergistic strategy integrating zincophilicity regulation, lattice matching, and 3D engineering is essential to construct a dynamically stable, highly reversible, and structurally robust electrode system for long-term operation under extreme conditions.
v. *Synergistic matching of Zn anode with other battery components*: Apart from Zn anode design, achieving high-performance AZIBs requires synergistic coordination with electrolytes and separators. Most current research focuses on the separate modification of individual battery components, while the synergistic optimization among the electrolyte solvation structure, the physicochemical properties of the Zn anode, and the separator functional design is often overlooked. For instance, the future strategy can integrate single-ion conducting separators with 3D hierarchically porous Zn anodes to enable the spatiotemporal synchronization of ion flux. Meanwhile, pairing specific alloy anodes with tailored electrolyte additives facilitates the synergistic construction of dynamic interphases. Through this holistic integration, the distinct functional advantages of individual components are synergistically amplified. Future research should establish a full-cell synergistic design system, where the intrinsic characteristics of each component are mutually matched, to realize comprehensive performance improvement of AZIBs (Fig. 11e).
vi. *Engineering fabrication of Zn anodes for high Zn utilization*: Practical commercial AZIBs require high areal capacity, a low negative-to-positive (N/P) capacity ratio, and high Zn utilization to achieve competitive energy density and cost efficiency. However, to increase the energy density of AZIBs, an unbalanced configuration is usually adopted, where the anode’s active mass is roughly 100 times that of the cathode, thereby limiting the practical Zn utilization below 1%. Future efforts should be directed toward the engineering fabrication of Zn anodes, developing low-cost, large-scale preparation methods that can maintain the anode’s structural stability and electrochemical performance under high Zn utilization conditions. This will accelerate the transition of Zn anodes from laboratory research to industrial practice (Fig. 11f).
